# Level of dietary energy and 2,4-thiazolidinedione alter molecular and systemic biomarkers of inflammation and liver function in Holstein cows

**DOI:** 10.1186/s40104-017-0196-y

**Published:** 2017-08-01

**Authors:** Afshin Hosseini, Mustafa Salman, Zheng Zhou, James K. Drackley, Erminio Trevisi, Juan J. Loor

**Affiliations:** 1 Department of Animal Sciences and Division of Nutritional Sciences, University of Illinois, 1207 West Gregory Drive, Urbana, IL 61801 USA; 2 Department of Animal Nutrition and Nutritional Diseases, University of Ondokuz Mayıs, 55139 Samsun, Turkey; 3 Istituto di Zootecnica, Facoltà di Scienze Agrarie Alimentari ed Ambientali, Università Cattolica del Sacro Cuore, 29122 Piacenza, Italy

**Keywords:** Inflammation, Insulin sensitivity, Nutrition, PPAR

## Abstract

**Background:**

The objective of the study was to evaluate the effect of overfeeding a moderate energy diet and a 2,4-thiazolidinedione (TZD) injection on blood and hepatic tissue biomarkers of lipid metabolism, oxidative stress, and inflammation as it relates to insulin sensitivity.

**Results:**

Fourteen dry non-pregnant cows were fed a control (CON) diet to meet 100% of NRC requirements for 3 wk, after which half of the cows were assigned to a moderate-energy diet (OVE) and half of the cows continued on CON for 6 wk. All cows received an intravenous injection of 4 mg TZD/kg of body weight (BW) daily from 2 wk after initiation of dietary treatments and for 2 additional week. Compared with CON cows and before TZD treatment, the OVE cows had lower concentration of total protein, urea and albumin over time. The concentration of cholesterol and tocopherol was greater after 2 wk of TZD regardless of diet. Before and after TZD, the OVE cows had greater concentrations of AST/GOT, while concentrations of paraoxonase, total protein, globulin, myeloperoxidase, and haptoglobin were lower compared with CON cows. Regardless of diet, TZD administration increased the concentration of ceruloplasmin, ROMt, cholesterol, tocopherol, total protein, globulin, myeloperoxidase and beta-carotene. In contrast, the concentration of haptoglobin decreased at the end of TZD injection regardless of diet. Prior to TZD injection, the mRNA expression of *PC*, *ANGPTL4*, *FGF21*, *INSR*, *ACOX1*, and *PPARD* in liver of OVE cows was lower compared with CON cows. In contrast, the expression of *HMGCS2* was greater in OVE compared with CON cows. After 1 wk of TZD administration the expression of *IRS1* decreased regardless of diet; whereas, expression of *INSR* increased after 2 wk of TZD injection. Cows fed OVE had lower overall expression of *TNF*, *INSR*, *PC*, *ACOX1*, *FGF21*, and *PPARD* but greater *HMGCS2* expression. These differences were most evident before and after 1 wk of TZD injection, and by 2 wk of TZD differences in expression for most genes disappeared.

**Conclusions:**

Based on molecular and blood data, administration of TZD enhanced some aspects of insulin sensitivity while causing contradictory results in terms of inflammation and oxidative stress. The bovine liver is TZD-responsive and level of dietary energy can modify the effects of TZD. Because insulin sensitizers have been proposed as useful tools to manage dairy cows during the transition period, further studies are required to investigate the potential hepatotoxicity effect of TZD (or similar compounds) in dairy cattle.

**Electronic supplementary material:**

The online version of this article (doi:10.1186/s40104-017-0196-y) contains supplementary material, which is available to authorized users.

## Background

Thiazolidinediones (TZD) are agonists of peroxisome proliferator-activated receptor gamma (PPARG) that elicit insulin-sensitizing effects in non-ruminants [1–3] and dairy cows [4–6]. The administration of TZD (4 mg of TZD/kg of BW) during the late prepartum period appeared to alter the dynamics of plasma glucose, non-esterified fatty acids (NEFA), and hydroxybutyric acid (BHBA) concentrations and dry matter intake (DMI) during the periparturient period [5, 7]. Furthermore, a greater concentration of blood insulin was reported in TZD-treated cows, which likely accounted for the lower NEFA [6].

We previously reported that in dry and non-pregnant dairy cows fed a control lower-energy (CON) or higher-energy (OVE) diet receiving 4 mg of TZD/kg of BW daily for 2 wk [8] the concentrations of glucose (4.55 vs. 4.65 mmol/L), insulin (27 vs. 35 μU/mL), and (BHBA) (0.27 vs. 0.37) were increased during the TZD administration (2 to 4 wk after diet initiation). In contrast, the concentration of NEFA (0.18 vs. 0.15 mmol/L) and adiponectin (ADIPOQ; 34.6 vs. 30.3 μg/L) remained unchanged during TZD administration. More importantly, the ratios of glucose/insulin (0.51 vs. 0.54) and NEFA/insulin (0.22 vs. 0.18) decreased during TZD, suggesting an improvement in insulin sensitivity. The mRNA expression in subcutaneous adipose tissue of *PPARG* and its targets *FASN* and *SREBF1*, which are the main regulators of adipogenesis and lipogenesis, were upregulated by TZD [8]. Greater expression of the insulin sensitivity-related genes *IRS1*, *SLC2A4*, *INSR*, *SCD*, *INSIG1*, *DGAT2*, and *ADIPOQ* in subcutaneous adipose tissue of OVE cows indicated that greater energy intake did not impair insulin sensitivity. In skeletal muscle, TZD altered expression of carbohydrate- and fatty acid oxidation-related genes. The OVE cows had greater mRNA expression of *PC* and *PCK1*, which was indicative of increased glyceroneogenesis [8].

A comprehensive review on the topic insulin sensitivity in dairy cattle underscored the role of skeletal muscle, adipose tissue, and liver in the overall glucose and insulin relationship [9]. The level of dietary energy fed prepartum has long been known to alter fat deposition and other metabolic pathways in tissues like liver [10]. Cows suffering from fatty liver had higher serum concentrations of NEFA, greater serum TNF, and had signs of systemic insulin resistance [11]. Some evidence indicates that excess NEFA concentrations alter hepatic function and inflammatory status [12, 13]. Importantly, the severity of inflammation around parturition seems related to some inflammatory indices observed in the dry period [14]. Thus, the available data seem to suggest that NEFA could cause inflammation and that inflammation also could cause the greater release of NEFA.

Our hypothesis was that overfeeding and TZD administration lead to changes in the dynamics of biomarkers of oxidative stress, inflammation, and metabolism in blood and liver tissue and might be related with systemic insulin sensitivity. To address the hypothesis, we measured in samples from Hosseini et al. [8] the plasma or serum concentrations of acute-phase proteins (haptoglobin, ceruloplasmin, albumin, cholesterol, and adiponectin), health and liver function biomarkers (total protein, myeloperoxidase, globulin, GGT, AST/GOT and total bilirubin), oxidative stress (paraoxonase and reactive oxygen metabolites (ROMt)), protein metabolism (urea), and vitamins (retinol, αtocopherol, beta-carotene). In addition, the mRNA expression of genes associated with glucose homeostasis and gluconeogenesis, lipid metabolism nuclear receptors and their targets, inflammation and ketogenesis was measured in liver tissue.

## Methods

### Experimental design, animals management and sampling

The Institutional Animal Care and Use Committee (IACUC) of the University of Illinois approved all procedures for this study (protocol #12134). Detailed materials and methods can be found elsewhere [8]. In brief, fourteen dry non-pregnant Holstein cows [initial BW (kg) = 717 ± 39; initial BCS = 3.31 ± 0.14] were assigned randomly to treatment groups. Cows were offered the TMR once daily at 0600 h and had unlimited access to fresh water. All cows were fed a control diet (CON; NE_L_ = 1.30 Mcal/kg) to meet 100% of NRC requirements for 3 wk, after which half of the cows were assigned to a moderate-energy diet (OVE; NE_L_ = 1.60 Mcal/kg) and half of the cows continued on CON for 6 wk. The OVE diet was fed ad libitum and resulted in cows consuming ~180% of NRC. Control cows were fed to consume only 100% of NRC. All cows received an intravenous injection of 4 mg TZD/kg of BW daily after the morning feeding into the jugular vein starting d 15 after the initiation of dietary treatments and until d 28. Blood samples were collected before the morning feeding from the coccygeal vein or artery every 5 ± 2 ds from −7 to 14 d of diet initiation (before TZD administration) and from 15 to 28 d of diet initiation (during TZD administration) for measurement of metabolites and hormones.

#### Biopsies, RNA isolation, primer design and evaluation, and quantitative PCR

Liver tissue was harvested via percutaneous biopsy as described previously [15] before the morning feeding at 14 d, before TZD administration, 21 and 28 d, during the TZD administration and 35 ds relative to diet initiation. Details of these procedures are reported in the supplemental material. Tissue was frozen immediately in liquid N until RNA extraction. The selection of the primers in liver was conducted base on core cellular functions, e.g. insulin signaling, glucose metabolism and synthesis, lipid metabolism, hepatokines, cytokines and inflammatory mediators (Additional file 1: Tables S1 and S2). The amplicons were sequenced and the fragment sequences were blasted and confirmed using the National Center for Biotechnology Information (NCBI). The sequences are shown in Additional file 1: Table S3. The geometric mean of internal control genes (ICG) *GAPDH*, ribosomal protein S9 (*RPS9*), and ubiquitously-expressed transcript (*UXT*) were used for data normalization (V2/3 ≤ 0.15) in liver tissue. qPCR perfromance is reported in Additional file 1: Table S4.

#### Statistical analysis

Data were analyzed with the Proc MIXED procedure of SAS 9.4 (SAS Institute Inc., Cary, NC). After normalization with the geometric mean of the ICG, the triplicate qPCR data for each gene were averaged and then log_2_ transformed prior to statistical analysis. Fixed effects in the model were diet, time, and diet × time. Cow within diet was designated as the random effect. Initial BW and BCS were included as covariates in the analysis for all variables, except when the covariate was not significant (*P* > 0.05). Blood metabolites were log-scale transformed if needed to comply with normal distribution of residuals, and means subsequently were back-transformed. Significance was declared at *P* ≤ 0.05, while trend was declared at *P* ≤ 0.10.

## Results

Results related to NE_L_ intake, BW, blood TZD as well as blood levels of BHBA, NEFA, glucose, insulin, adiponectin were reported previously [8].

### Blood biomarkers before TZD administration

**Fig. 1:**
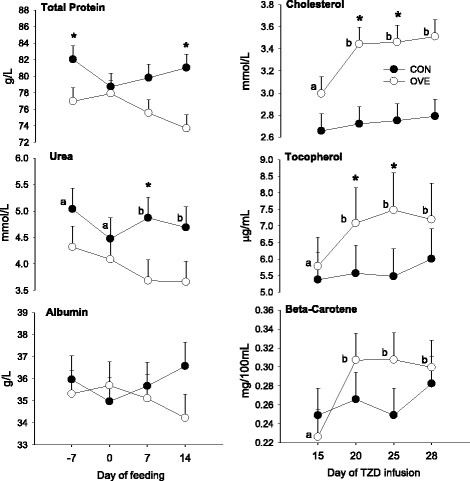
Temporal concentrations [−7 to 14 d relative to diet initiation; before TZD administration and 15 to 28 d relative to diet initiation (during TZD administration)] of blood biomarkers in cows fed either a controlled-energy diet (CON, 1.30 Mcal/kg diet dry matter; *n* = 7) or a moderate-energy diet (OVE, 1.60 Mcal/kg diet dry matter; *n* = 7) before (*left panels*) and during (*right panels*) 2,4-thiazolidinedione (TZD) administration. *Significant (*P* < 0.05) difference due to diet or Diet × Time. ^a,b^Significant (*P* < 0.05) overall difference due to time

The OVE cows had lower (*P* ≤ 0.05) concentration over time of total protein, urea, and albumin (Fig. 1). The overall concentration of cholesterol and tocopherol increased (*P* < 0.05) at 14 d after diet initiation, whereas the serum concentration of total bilirubin tended (*P* = 0.10) to decrease at 14 d after diet initiation (Table 1). Prior to TZD, diet did not affect (*P* > 0.05) cholesterol, total bilirubin, or tocopherol. Serum concentrations of glucose, ceruloplasmin, ROMt, GGT, retinol, AST/GOT, paraoxonase, globulin, myeloperoxidase, haptoglobin, and beta-carotene also were not affected (*P* > 0.05, data not shown) by diet, time (14 wk) or their interaction.

**Table 1:** Plasma concentrations of biomarkers from −7 to 14 d relative to diet initiation, before 2,4-thiazolidinedione (TZD) administration and 15 to 28 d relative to diet initiation and during TZD administration in cows fed either a controlled-energy diet (CON, 1.30 Mcal/kg; *n* = 7) or a moderate-energy diet (OVE, 1.60 Mcal/kg; *n* = 7)

Item	Diet			Day					*P*-value	
	CON	OVE	SEM	−7	0	7	14	SEM	Diet	Day
Before TZD				−	−	−	−			
Metabolism										
Cholesterol, mmol/L	2.64	2.68	0.15	2.62^a^	2.54^a^	2.64^a^	2.84^b^	0.11	0.86	< 0.01
Liver function										
Total Bilirubin, μU/mL	1.18	1.06	0.10	1.19^c^	1.09^d^	1.11^c^	1.08^d^	0.09	0.14	0.10
Antioxidants–anti-inflammation										
α-Tocopherol, mmol/L	5.22	5.28	0.46	5.19^a^	5.02^a^	5.13^a^	5.65^b^	0.34	0.94	< 0.01
Item	Diet			Day					*P*-value	
	CON	OVE	SEM	15	20	25	28	SEM	Diet	TZD
During TZD				+	+	+	+			
Health and liver function										
GGT, U/L	22.7	21.3	2.8	21.3^c^	22.2^cd^	21.6^cd^	22.9^d^	2.1	0.53	0.07
Globulin, g/L	43.6^b^	38.8^a^	1.0	39.8^a^	42.0^b^	41.5 ^b^	41.5^b^	1.0	0.03	0.02
Myeloperoxidase, U/L	491^b^	419^a^	21.5	399^a^	474^b^	484^b^	462^b^	24.1	0.03	0.05
Total Protein, g/L	78.7^b^	73.0^a^	1.2	74.1^a^	77.2^b^	76.0 ^b^	76.2^b^	1.0	< 0.01	0.021
Total Bilirubin, μmol/L	1.19^d^	1.03^c^	0.11	1.48	1.39	1.39	1.37	0.42	0.06	0.97
Acute-phase proteins										
Ceruloplasmin, μmol/L	3.30	3.14	0.11	2.95^a^	3.30^b^	3.30^b^	3.34^b^	0.10	0.33	< 0.01
Haptoglobin, g/L	0.65^b^	0.51^a^	0.05	0.62^a^	0.57^a,b^	0.61^a^	0.51^b^	0.068	< 0.01	0.05
Oxidative stress										
ROMt, mg H_2_O_2_/100 mL	13.8	13.4	0.57	12.1^a^	14.0^b^	14.1^b^	14.2^b^	0.51	0.62	< 0.01
Paraoxonase, U/mL	113^b^	91.4^a^	6.4	100	104	99.8	105	5.14	0.03	0.15
Liver injury										
AST/GOT, U/L	66.0^a^	77.6^b^	3.6	67.8	72.8	75.8	70.8	3.3	0.04	0.16
Antioxidants–anti-inflammation										
Retinol, μg/100 mL	37.8^c^	47.6^d^	3.7	42.6	43.5	42.9	41.8	2.77	0.09	0.56

^a,b^Means within a row with different superscripts differ (*P* ≤ 0.05) for TZD, while ^c,d^ denote trends (*P* ≤ 0.10)

### Blood biomarkers and hormones during TZD administration

The Fig. 1 and Table 1 contain the main effects of diet, TZD, and interactions for plasma parameters. Although plasma cholesterol did not differ statistically prior to TZD (Table 1), during TZD there was greater (interaction *P* < 0.05) concentration of cholesterol and tocopherol in cows fed OVE (Fig. 1). Regardless of diet, the TZD increased (*P* < 0.05) GGT, ceruloplasmin, and ROMt concentrations at d 28 from diet initiation (Table 1). An interaction during TZD was detected for concentrations of globulin, myeloperoxidase, total protein, and haptoglobin due to lower overall concentrations in cows fed OVE. Independent of TZD, the concentrations of total bilirubin and paraoxonase were lower and AST/GOT greater in cows fed OVE (Table 1). No effect of diet, TZD or their interaction (*P* > 0.05) was detected for urea and albumin concentrations (Fig. 1).

### Gene expression in response to level of diet and TZD

**Fig. 2:**
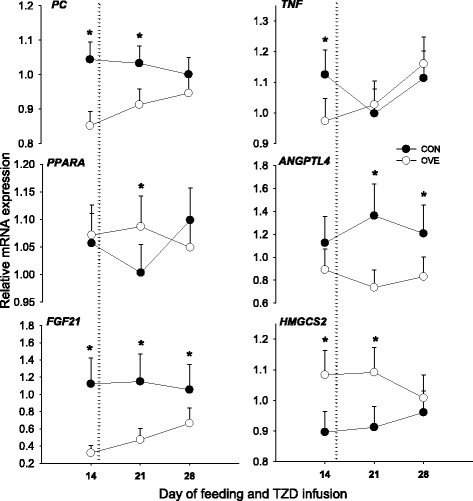
Expression of genes associated with gluconeogenesis (*PC*), lipid metabolism nuclear receptors (*PPARA*), ketogenesis (*HMGCS2*), hepatokines (*ANGPTL4* and *FGF21*)*,* and inflammation (*TNF*) in liver tissue of cows fed either a controlled-energy diet (CON, 1.30 Mcal/kg diet dry matter; *n* = 7) or a moderate-energy diet (OVE, 1.60 Mcal/kg diet dry matter; *n* = 7) before and during 2,4-thiazolidinedione (TZD) administration. The *dotted line* denotes the start of TZD infusion on d 15 relative to diet initiation. *Significant (*P* < 0.05) difference due to diet or Diet × Time. ^a,b^Significant (*P* < 0.05) overall difference due to time

The Fig. 2 and Table 2 contain the main effects of diet, TZD, and interactions for genes related to insulin signaling, glucose metabolism, fatty acid oxidation, hepatokines, ketogenesis and inflammation. Prior to and during TZD, the expression of *ACOX1* and *PPARD* was lower (*P* < 0.05) in cows fed OVE (Table 2). In OVE cows, the mRNA expression of *PC*, *FGF21*, and *INSR* was lower (*P* < 0.05), while the mRNA expression of *HMGCS2* was greater (*P* < 0.05) during TZD (Fig. 2 and Table 2). Regardless of diet, the TZD administration increased (*P* < 0.05) the mRNA of *IRS1* and *TNF* at 28 d after diet initiation (Fig. 2 and Table 2).

**Table 2:** Relative mRNA expression of target genes in liver tissue at 14 to 28 d relative to diet initiation and after a 7-day washout period (35 d) in cows fed a controlled-energy diet (CON, 1.30 Mcal/kg; *n* = 7) or a moderate-energy diet (OVE, 1.60 Mcal/kg; *n* = 7). All cows received an intravenous injection of 2,4-thiazolidinedione (TZD) (+) for 2 wk after biopsy collection at 14 d after initiation of dietary treatments, with the additional 2 wk of study serving as a washout period

Gene	Diet			Day					*P*-value	
	CON	OVE	SEM	14	21	28	35	SEM	Diet	TZD
TZD				−	+	+	−			
*IRS1*	1.03	1.02	0.03	1.04^a^	0.99^b^	1.03^a^	-^c^	0.029	0.77	0.04
*INSR*	1.12^b^	1.02^a^	0.05	1.06^a^	1.04^a^	1.11^b^	−	0.04	0.02	0.03
*ACOX1*	1.03^b^	0.96^a^	0.02	1.01	0.98	1.00	−	0.02	< 0.01	0.16
*PPARD*	1.07^b^	0.99^a^	0.04	1.04	1.01	1.03	−	0.03	0.01	0.21

^a,b^Means within a row with different superscripts differ (*P* ≤ 0.05) due to diet or TZD

^c^Tissue sample not available

## Discussion

The ruminant liver is the main site for gluconeogenesis [16], and a spike of insulin into the bloodstream suppresses not only gluconeogenesis but also glycogenolysis and AT tissue lipolysis [9]. In fact, elevated insulin concentrations can stimulate glycogenesis in liver and improve overall insulin sensitivity via glucose uptake in peripheral tissues such as AT and skeletal muscle [9]. In humans with type 2 diabetes, the injection of TZD decreased hepatic fat content and increased hepatic insulin sensitivity along with increased glucose uptake by peripheral tissues [17]. Thus, despite inherent differences in gastrointestinal tract anatomy, circulating insulin can alter insulin sensitivity in extrahepatic tissues in ruminants and non-ruminants.

Besides its effect on insulin sensitivity, previous data from non-ruminants also indicated that TZD changes the dynamics of blood biomarkers, e.g. adiponectin, insulin and glucose. Similarly, in dairy cows the administration of TZD (4 mg of TZD/kg of BW) during the late-prepartum period altered the dynamics of plasma glucose, NEFA, BHBA concentrations and also DMI during the periparturient period. The TZD helped cows to maintain BCS postpartum [5, 7] and achieve greater concentrations of insulin [6], which likely accounted for the lower NEFA, i.e. the drug probably induced greater insulin sensitivity in peripheral tissues such as AT. In the present study the TZD administration increased triacylglycerol concentration and altered expression of carbohydrate- and fatty acid oxidation-related genes in skeletal muscle [8]. Furthermore, the upregulation of *PPARG* with the TZD injection provided evidence that TZD could enhance adipogenesis and lipogenesis in SAT, while differentially regulating glucose homeostasis and fatty acid oxidation in skeletal muscle [8].

### Diet, TZD and blood biomarkers

#### Overfeeding energy intake and hepatic function

The lower blood concentration of urea in OVE cows was in accordance with previous results [18]. A better liver function status in OVE cows is supported by the lower total bilirubin [12, 13]. In addition, the hyperinsulinemia in OVE cows [8] along with lower urea concentration also might be related to increased gluconeogenesis in liver along with amino acid uptake and protein synthesis in skeletal muscle [19].

In addition to its well-described role as a negative acute-phase protein (negAPP), the rate of albumin synthesis is affected by feed intake [20–22], i.e. greater concentrations are correlated with higher energy intake but in the absence of differences in energy intake a lower concentration is indicative of altered liver function. The difference between the total protein and albumin content in plasma provides an estimate of globulin concentration [13]. The dynamics of the change in globulin concentration is considered an index of immunoglobulin production [12]. Thus, as indicated by the present data, the mild decrease in total protein and albumin concentrations in OVE despite the greater DMI [8] is suggestive of a possible impairment in synthesis at the liver level brought about by a mild inflammation. However, the more pronounced decrease globulin seems to argue against inflammation playing a role. These results are in accordance with some previous studies in energy-overfed dairy cows [18]. Whether excess energy intake caused a mild chronic degree of inflammation [23] remains to be determined.

The sustained decrease in plasma concentrations of cholesterol before calving and the weak increase after calving have been associated with an inflammatory-like status [12, 13], but also reflect the pattern of DMI, i.e. gradual decrease through calving followed by a gradual increase. Clearly, the response in the present study could not be compared with the periparturient period where negative energy balance is a consequence of higher energy requirements for milk production rather and lower DMI. Thus, the greater blood concentration of cholesterol in OVE cows is likely a reflection of the greater DMI, i.e. cholesterol synthesized by the intestine [24]. A review of the literature [25] concluded that inflammation could alter lipid transport in the circulation through changes in the dynamics of apolipoproteins.

Paraoxonase is considered an antioxidative stress enzyme and a negAPP that binds to plasma apolipoproteins [26]. An antioxidative function of paraoxonase has been suggested in dairy cows [27], as well as a link with the dynamics of positive acute-phase proteins (posAPP) and negAPP around parturition [12]. In humans, the serum paraoxonase activity is reduced by oxidized low density lipoprotein (LDL) and is maintained or increased by antioxidants [28]. Reactive oxygen metabolites (ROMt) are considered useful biomarkers of oxidative stress; the results of an in vivo study in mice and an in vitro study in human hepatocyte cell cultures revealed that concentrations of ROMt can be increased by feeding high-carbohydrate diets [29]. Those studies revealed that the increase in ROMt decreased the capacity of hepatocytes to respond to an oxidative challenge. In our study, the lower concentration of paraoxonase coupled with greater cholesterol in OVE cows indicated a reduction in the ability of the liver to synthesize this enzyme. In fact, work with dairy cows has revealed a positive relationship between increases in plasma cholesterol and paraoxonase, which underscores the idea that liver function in OVE cows experienced a negative effect for some functional aspects.

The liver produces the major portion of the systemic haptoglobin in mammals, but in humans its concentration has been used as a marker for adiposity [30]. In bovine, however, haptoglobin is a major posAPP and its concentration in plasma is increased during inflammation, infection, and often during the transition period [31]. Despite the marked increased in energy intake, the lower concentration of haptoglobin in OVE cows likely reflects a lower inflammatory and oxidative stress response.

Regardless of TZD, the overall blood concentrations of cholesterol, albumin, and total protein were within a non-pathologic range [32]. Along with the lower concentration of paraoxonase, haptoglobin, and myeloperoxidase, the greater concentrations of cholesterol in OVE cows indicated the absence of important impairment of the synthesis and release of the lipoproteins, and also of a severe acute-phase reaction in the liver. Furthermore, the lower bilirubin in OVE cows indicated normal hepatic bilirubin clearance capacity and agrees with the lower inflammatory status [32]. Despite all the positive systemic signs in OVE cows, they had greater concentration of AST/GOT, which in clinical studies is an indicator of liver tissue damage [33]. However, the concentration of AST/GOT can be affected by catabolism of nutrients such as amino acids via the citric acid cycle [34]. Thus, the greater AST/GOT in OVE cows might be related not only to “leakage” from liver cells but also to greater citric acid cycle flux, e.g. to produce glucose, as a result of the greater DMI and the greater absorption of amino acids from the gut.

The greater blood concentrations of α-tocopherol, retinol, and beta-carotene in OVE cows is suggestive of adequate or better vitamin status, and these molecules have been linked with the immune response. Beta-carotene and α-tocopherol are antioxidants that bind free radicals to prevent lipid peroxidation and improve the immune response [35, 36]. Retinol (vitamin A) circulates in the blood bound to retinol-binding protein (RBP), a protein that is synthesized in the liver and controls availability of the vitamin in times of insufficient dietary intake [37]. Inhibition of RBP transcription or function is one determinant of circulating levels of retinol. Similar to retinol, the liver is considered the “master regulator” of systemic vitamin E availability, i.e. it controls disposition, metabolism, and excretion [38]. In the context of lipoprotein metabolism, re-packing of α-tocopherol into the very-low density lipoproteins is central for extra-hepatic availability. Thus, hepatic metabolism of cholesterol and lipoproteins clearly has direct bearing on systemic α-tocopherol concentrations. In the context of insulin sensitivity, the greater overall concentration of retinol in cows fed OVE could have had an effect on peripheral insulin sensitivity as demonstrated recently [39]. These relationships likely explain the greater α-tocopherol concentration in OVE cows which also had greater cholesterol concentration (i.e. an index of lipoprotein synthesis). Besides dietary availability, in non-ruminants, the acute-phase response causes a decrease in the hepatic synthesis of vitamin A and of α-tocopherol namely due to alterations in hepatic function [40]. Clearly, the greater blood concentration of these vitamins especially retinol in OVE cows was in part due to the greater DMI and diet composition, and the resulting storage of these vitamins is not expected to impair liver function [41].

#### TZD, insulin sensitizing effect versus Hepatotoxicity

Historically, TZD has been used over the last two decades to improve insulin sensitivity in type II diabetic subjects. The results of in vivo and in vitro studies [42, 43] indicated that Troglitazone (TGZ), a thiazolidinedione, caused hepatotoxicity through non-metabolic and metabolic factors [44]. Some of the present data could reflect a mild hepatotoxic effect of the short-term TZD administration. For instance, in addition to the decrease in the negAPP (e.g. cholesterol), there seems to have been differential regulation of the posAPP by TZD, e.g. ceruloplasmin increased and haptoglobin decreased. Results from a previous study revealed that TZD can decrease haptoglobin expression in adipocytes in vitro and in vivo [45]. Because the cows were clinically healthy during the study, it is also possible that the greater concentration of ceruloplasmin after TZD regardless of diet was associated with a stressful effect also reflected by the gradual increase in ROMt with TZD regardless of diet.

There is evidence from rats [46] and humans [47] that the family of TZD compounds might cause oxidative stress and elicit a direct effect on mitochondrial physiology, hence, playing a role in TZD-mediated hepatotoxicity. In our study, the administration of TZD increased ROMt concomitantly with GGT, AST/GOT, myeloperoxidase, globulin and total protein. A study in mice reported that insulin-sensitizing compounds may act as antioxidants while increasing oxidative stress in the liver [42]. A recent study detected an increase in the synthesis of liver enzymes such as AST and ALT in Chinese subjects with type II diabetic using 4 mg of a TZD-like compound for three month. The mechanistic reasons for elevated liver enzymes upon TZD treatment remain to be determined.

The blood concentration of cholesterol has been used as an indicator of lipoprotein metabolism and is linked to the quantity of lipid mobilized and re-esterified as VLDL within the intestine and liver [32]. In the present study, the greater cholesterol concentration during TZD might indicate the increased export of VLDL to peripheral tissues such as adipose and skeletal muscle. We speculate that such response would have provided insulin-sensitive tissues with fatty acids for esterification, an idea supported by the greater concentration of triacylglycerol that was detected in muscle during TZD injection [8]. Similar to cholesterol, it is possible that the greater concentration of α-tocopherol is a reflection of an increase in VLDL export from the liver [48] to peripheral tissues during the TZD injection period.

#### TZD modulates hepatic metabolic pathways via *PPARA* and its targets

The state of systemic insulin sensitivity or glucose tolerance can be estimated using the euglycemic clamp [49] and glucose tolerance tests [4]. In our study, the OVE cows had a greater overall concentration of insulin [8], thus, the lower hepatic expression of *INSR* in the liver was somewhat expected [50]. Although it is well-established that the ruminant liver is not a net user of glucose, our results of insulin signaling-related genes are in accordance with previous results in rodents. For instance, a hyperinsulinemic state in obese mice led to a decrease in insulin binding and phosphorylation of the insulin receptor and the kinase IRS-1 in liver [51]. The data seem to agree with studies in sheep demonstrating differences in the binding of insulin to receptors in liver as a function of circulating concentration [50]. Furthermore, our data indicate that TZD could increase insulin sensitivity and signaling in cow liver [52].

Comprehensive reviews of available data have concluded that PPARα is the main regulator of hepatic fatty acid oxidation, ketogenesis, triglyceride turnover, and gluconeogenesis in non-ruminants and likely in ruminants [53, 54]. For example, the results of studies with *PPARA* knockout mice and bovine cells provided strong evidence that gluconeogenesis and glycerol metabolism are directly controlled by PPARA [55] via its targets PC and PCK1 [56, 57]. The temporal profile of *PC* in the present study seems to suggest the existence of an alternate mechanism whereby insulin sensitizers (over the long-term) could affect hepatic gluconeogenesis differently in response to dietary energy level, i.e. overfeeding moderate energy diets over a period of weeks seems to render the liver less responsive to TZD while feeding to meet energy requirements cannot prevent the expected negative effect of TZD on insulin sensitivity [58]. The exact mechanisms for the TZD effect merit further study.

In ruminants, there is indirect evidence that long-chain fatty acids (LCFA) can activate hepatic β-oxidation via PPARA and its downstream co-activators to decrease tissue TAG accumulation [53]. ACOX1 is one of the PPARA targets which is involved in peroxisomal fatty acid oxidation in non-ruminants; it is the first enzyme in peroxisomal LCFA oxidation [59, 60]. A lower *ACOX1* expression in OVE cows could be explained by the lower plasma NEFA compared with CON cows. In the present study, the different regulation of mRNA expression of proximal and outer-mitochondrial β-oxidation enzymes by TZD administration might have been related with the fact that this compound binds to mitochondrial membrane proteins to modulate mitochondrial metabolism [61].

Research in non-ruminants has demonstrated that ANGPTL4 [62] and FGF21 [63, 64] are the targets of PPARA, and are considered hepatokines, i.e. proteins secreted from liver that can regulate tissue adaptations to feed restriction [65, 66]. A study with mouse primary hepatocytes underscored the negative effect of insulin on mRNA expression of *ANGPTL4* [67]. In rodents, fasting and starvation activated ANGPTL4 in a PPARA-dependent manner [68]; the study of Loor et al. [65] uncovered a potential link between PPARA and ANGPTL4 in cows during nutrition-induced ketosis. ANGPTL4 is an endogenous inhibitor of lipoprotein lipase (LPL) that regulates peripheral tissue uptake of plasma TAG-derived fatty acids [69], hence, helping to regulate energy homeostasis.

In vitro studies in rodents have revealed that PPAR agonists such as TZD are potent inducers of FGF21 [70]. In diabetic rodents and monkey, FGF21 is considered an effective metabolic regulator of glucose and lipid homeostasis in the context of insulin resistance, glucose intolerance and dyslipidemia [68]. Additional studies indicated an increase in plasma concentration and expression of FGF21 during the transition period [66].

In the present study, the lower expression of *ANGPTL4* and *FGF21* in OVE agrees with the fact they were in positive energy balance. Furthermore, a lower *FGF21* expression would be expected in OVE cows because they had higher blood insulin concentration [8]. The upregulation of *FGF21* mRNA expression at two weeks. After TZD administration agrees with a mouse study in which the TZD induced the expression of this gene [70]. Our data indicate that bovine *PPARA* is TZD responsive, and may participate in the regulation of hepatic insulin sensitivity and energy expenditure via its targets. The lower *PPARD* mRNA expression in OVE cows might be related to the lower plasma NEFA concentration; in an in vitro study with rodents, an upregulation of *PPARD* was determined to be important in order to “sense” concentrations of LCFA as a way to control mitochondrial oxidation in order to protect against fatty acid-induced cellular dysfunction.

The HMGCS2 enzyme is rate-limiting for the production of ketone bodies in liver and the neonatal intestine [71]. In growing ruminants, the mRNA expression of *HMGCS2* also is associated with ketogenesis in ruminal epithelium, a process that helps the rumen develop [72]. Studies with rodents uncovered that the expression of *HMGCS2* is regulated by PPARA or by conditions that enhance LCFA influx into the liver (e.g. feed restriction) [73]. In contrast, insulin signaling in liver is a negative regulator of *HMGCS2* expression and activity. Thus, in the present study the greater mRNA expression of *HMGCS2* in OVE cows might have been linked with a lower plasma insulin concentration. In contrast, the gradual downregulation of *HMGCS2* expression during the TZD injection period could have been associated with an insulin-sensitizing effect.

## Conclusions

Despite a marked increase in energy balance status in OVE cows in response to overfeeding energy there were modest alterations in the acute-phase response, but it should be kept in mind that the linear decrease in albumin from day 0 to 14 is an indication of a gradual impairment of some liver functions. Based on molecular and blood data it seems that administration of TZD enhanced some aspects of insulin sensitivity while causing contradictory results in terms of inflammation and oxidative stress. Overall, data indicated that bovine liver is TZD responsive and level of dietary energy can modify the effects of TZD. Because insulin sensitizers have been proposed as useful tools to manage dairy cows during the transition period, further studies are required to investigate the potential hepatotoxicity effect of TZD (or similar compounds) in dairy cattle.

## Additional file

Additional file 1: Table S1. GenBank accession number, sequence and amplicon size of primers used to analyze gene expression by quantitative PCR. **Table S2** Gene symbol, gene name, and description of the main biological function and biological processes of the targets analyzed in subcutaneous adipose tissue and muscle. **Table S3** Sequencing results obtained from PCR product. **Table S4** qPCR performance among the genes measured in adipose tissue and muscle. (DOCX 197 kb)

## Abbreviations

ACD: Citric acid and dextrose
DIM: Days in milk
DMI: Dry matter intake
IACUC: Institutional animal care and use committee
PPAR: Peroxisome proliferator-activated receptors
ROMt: Reactive oxygen metabolites

## References

[1] Ahmadian M, Suh JM, Hah N, Liddle C, Atkins AR, Downes M (2013). PPARgamma signaling and metabolism: the good, the bad and the future. Nat Med.

[2] Jonas D, Van Scoyoc E, Gerrald K, Wines R, Amick H, Triplette M, et al. in Drug Class Review: Newer Diabetes Medications, TZDs, and Combinations. Final Original Report. 2011. Oregon Health & Science University, Portland (OR). 21595121

[3] Ye J. Challenges in drug discovery for Thiazolidinedione substitute. Acta Pharmaceutica Sinica B. 2011;1(3):137–42. 10.1016/j.apsb.2011.06.011 PMC3302162 22427718

[4] Schoenberg KM, Overton TR (2011). Effects of plane of nutrition and 2,4-thiazolidinedione on insulin responses and adipose tissue gene expression in dairy cattle during late gestation. J Dairy Sci.

[5] Smith KL, Butler WR, Overton TR (2009). Effects of prepartum 2,4-thiazolidinedione on metabolism and performance in transition dairy cows. J Dairy Sci.

[6] Smith KL, Stebulis SE, Waldron MR, Overton TR (2007). Prepartum 2,4-thiazolidinedione alters metabolic dynamics and dry matter intake of dairy cows. J Dairy Sci.

[7] Schoenberg KM, Perfield KL, Farney JK, Bradford BJ, Boisclair YR, Overton TR (2011). Effects of prepartum 2,4-thiazolidinedione on insulin sensitivity, plasma concentrations of tumor necrosis factor-alpha and leptin, and adipose tissue gene expression. J Dairy Sci.

[8] Hosseini A, Tariq MR, Trindade da Rosa F, Kesser J, Iqbal Z, Mora O (2015). Insulin sensitivity in adipose and skeletal muscle tissue of dairy cows in response to dietary energy level and 2,4-Thiazolidinedione (TZD). PLoS One.

[9] De Koster JD, Opsomer G (2013). Insulin resistance in dairy cows. Vet Clin North Am Food Anim Pract.

[10] Khan MJ, Jacometo CB, Graugnard DE, Corrêa MN, Schmitt E, Cardoso F, et al. Overfeeding dairy cattle during late-pregnancy alters hepatic PPARalpha-regulated pathways including Hepatokines: impact on metabolism and peripheral insulin sensitivity. Gene Regul Syst Bio. 2014;8:97–111. 10.4137/GRSB.S14116 PMC3981572 24737933

[11] Ohtsuka H, Koiwa M, Hatsugaya A, Kudo K, Hoshi F, Itoh N (2001). Relationship between serum TNF activity and insulin resistance in dairy cows affected with naturally occurring fatty liver. J Vet Med Sci.

[12] Bionaz M, Trevisi E, Calamari L, Librandi F, Ferrari A, Bertoni G (2007). Plasma paraoxonase, health, inflammatory conditions, and liver function in transition dairy cows. J Dairy Sci.

[13] Bertoni G, Trevisi E, Han X, Bionaz M (2008). Effects of inflammatory conditions on liver activity in puerperium period and consequences for performance in dairy cows. J Dairy Sci.

[14] Trevisi E, Zecconi A, Bertoni G, Piccinini R (2010). Blood and milk immune and inflammatory profiles in periparturient dairy cows showing a different liver activity index. J Dairy Res.

[15] Dann HM, Litherland NB, Underwood JP, Bionaz M, D'Angelo A, McFadden JW, et al. Diets during far-off and close-up dry periods affect periparturient metabolism and lactation in multiparous cows. J Dairy Sci. 2006;89(9):3563–77. 10.3168/jds.S0022-0302(06)72396-7 16899692

[16] Bas P (1992). Changes in activities of lipogenic enzymes in adipose tissue and liver of growing goats. J Anim Sci.

[17] Chang E, Park CY, Park SW (2013). Role of thiazolidinediones, insulin sensitizers, in non-alcoholic fatty liver disease. J Diabetes Investig.

[18] Graugnard DE, Bionaz M, Trevisi E, Moyes KM, Salak-Johnson JL, Wallace RL, et al. Blood immunometabolic indices and polymorphonuclear neutrophil function in peripartum dairy cows are altered by level of dietary energy prepartum. J Dairy Sci. 2012;95(4):1749–58. 10.3168/jds.2011-4579 22459823

[19] Bolster DR, Jefferson LS, Kimball SR (2004). Regulation of protein synthesis associated with skeletal muscle hypertrophy by insulin-, amino acid- and exercise-induced signalling. Proc Nutr Soc.

[20] Kirsch R, Frith L, Black E, Hoffenberg R (1968). Regulation of albumin synthesis and catabolism by alteration of dietary protein. Nature.

[21] Nicholson JP, Wolmarans MR, Park GR (2000). The role of albumin in critical illness. Br J Anaesth.

[22] Lunn PG, Austin S (1983). Dietary manipulation of plasma albumin concentration. J Nutr.

[23] De Matteis L, Bertoni G, Lombardelli R, Wellnitz O, Van Dorland HA, Vernay MCMB, et al. Acute phase response in lactating dairy cows during hyperinsulinemic hypoglycaemic and hyperinsulinemic euglycaemic clamps and after intramammary LPS challenge. J Anim Physiol Anim Nutr (Berl). 2017;101(3):511–20. 10.1111/jpn.12463 27079943

[24] Duske K, Hammon HM, Langhof AK, Bellmann O, Losand B, Nürnberg K, et al. Metabolism and lactation performance in dairy cows fed a diet containing rumen-protected fat during the last twelve weeks of gestation. J Dairy Sci. 2009;92(4):1670–84. 10.3168/jds.2008-1543 19307649

[25] Katoh N (2002). Relevance of apolipoproteins in the development of fatty liver and fatty liver-related peripartum diseases in dairy cows. J Vet Med Sci.

[26] Mackness B, Durrington PN, Mackness MI (1998). Human serum paraoxonase. Gen Pharmacol.

[27] Turk R, Juretić D, Geres D, Svetina A, Turk N, Flegar-Mestrić Z (2008). Influence of oxidative stress and metabolic adaptation on PON1 activity and MDA level in transition dairy cows. Anim Reprod Sci.

[28] Aviram M, Rosenblat M, Billecke S, Erogul J, Sorenson R, Bisgaier CL, et al. Human serum paraoxonase (PON 1) is inactivated by oxidized low density lipoprotein and preserved by antioxidants. Free Radic Biol Med. 1999;26(7–8):892–904. 10.1016/s0891-5849(98)00272-x 10232833

[29] Collison KS, Saleh SM, Bakheet RH, Al-Rabiah RK, Inglis AL, Makhoul NJ, et al. Diabetes of the liver: the link between nonalcoholic fatty liver disease and HFCS-55. Obesity (Silver Spring). 2009;17(11):2003–13. 10.1038/oby.2009.58 19282820

[30] Chiellini C, Santini F, Marsili A, Berti P, Bertacca A, Pelosini C, et al. Serum haptoglobin: a novel marker of adiposity in humans. J Clin Endocrinol Metab. 2004;89(6):2678–83. 10.1210/jc.2003-031965 15181041

[31] Ceciliani F, Ceron JJ, Eckersall PD, Sauerwein H (2012). Acute phase proteins in ruminants. J Proteome.

[32] Bertoni G, Trevisi E (2013). Use of the liver activity index and other metabolic variables in the assessment of metabolic health in dairy herds. Vet Clin North Am Food Anim Pract.

[33] Kaneko JJ, Harvey JW, Bruss ML (1997). Clinical biochemistry of domestic animals.

[34] Giannini EG, Testa R, Savarino V (2005). Liver enzyme alteration: a guide for clinicians. CMAJ.

[35] Bendich A (1993). Physiological role of antioxidants in the immune system. J Dairy Sci.

[36] LeBlanc SJ, Herdt TH, Seymour WM, Duffield TF, Leslie KE (2004). Peripartum serum vitamin E, retinol, and beta-carotene in dairy cattle and their associations with disease. J Dairy Sci.

[37] Noy N (2016). Vitamin a in regulation of insulin responsiveness: mini review. Proc Nutr Soc.

[38] Traber MG (2007). Vitamin E regulatory mechanisms. Annu Rev Nutr.

[39] Berry DC, Noy N (2012). Signaling by vitamin a and retinol-binding protein in regulation of insulin responses and lipid homeostasis. Biochim Biophys Acta.

[40] Gruys E, Toussaint MJ, Niewold TA, Koopmans SJ (2005). Acute phase reaction and acute phase proteins. J Zhejiang Univ Sci B.

[41] Wolf G (1984). Multiple functions of vitamin a. Physiol Rev.

[42] Kassahun K, Pearson PG, Tang W, McIntosh I, Leung K, Elmore C, et al. Studies on the metabolism of Troglitazone to reactive intermediates in vitro and in vivo. Evidence for novel biotransformation pathways involving Quinone Methide formation and Thiazolidinedione ring scission. Chem Res in Toxicol. 2001;14(1):62–70. 10.1021/tx000180q 11170509

[43] Hu D, Wu CQ, Li ZJ, Liu Y, Fan X, Wang QJ, et al. Characterizing the mechanism of thiazolidinedione-induced hepatotoxicity: an in vitro model in mitochondria. Toxicol Appl Pharmacol. 2015;284(2):134–41. 10.1016/j.taap.2015.02.018 25727309

[44] Masubuchi Y (2006). Metabolic and non-metabolic factors determining Troglitazone Hepatotoxicity: a review. Drug Metab Pharmacokinet.

[45] Vernochet C, Davis KE, Scherer PE, Farmer SR (2010). Mechanisms regulating repression of haptoglobin production by peroxisome proliferator-activated receptor-gamma ligands in adipocytes. Endocrinology.

[46] Narayanan PK, Hart T, Elcock F, Zhang C, Hahn L, McFarland D, et al. Troglitazone-induced intracellular oxidative stress in rat hepatoma cells: a flow cytometric assessment. Cytometry A. 2003;52(1):28–35. 10.1002/cyto.a.10011 12596249

[47] Garcia-Ruiz I, Rodríguez-Juan C, Díaz-Sanjuán T, Martínez MA, Muñoz-Yagüe T, Solís-Herruzo JA (2007). Effects of rosiglitazone on the liver histology and mitochondrial function in ob/ob mice. Hepatology.

[48] Cohn W, Loechleiter F, Weber F (1988). Alpha-tocopherol is secreted from rat liver in very low density lipoproteins. J Lipid Res.

[49] Petterson JA, Dunshea FR, Ehrhardt RA, Bell AW (1993). Pregnancy and undernutrition alter glucose metabolic responses to insulin in sheep. J Nutr.

[50] Gill RD, Hart IC (1980). Properties of insulin and glucagon receptors on sheep hepatocytes: a comparison of hormone binding and plasma hormones and metabolites in lactating and non-lactating ewes. J Endocrinol.

[51] Saad MJ, Saad MJ, Araki E, Miralpeix M, Rothenberg PL, White MF, et al. Regulation of insulin receptor substrate-1 in liver and muscle of animal models of insulin resistance. J Clin Invest. 1992;90(5):1839–49. 10.1172/JCI116060 PMC443244 1331176

[52] Smith DH, Palmquist DL, Schanbacher FL (1986). Characterization of insulin binding to bovine liver and mammary microsomes. Comp Biochem Physiol A Comp Physiol.

[53] Bionaz M, Chen S, Khan MJ, Loor JJ (2013). Functional role of PPARs in ruminants: potential targets for fine-tuning metabolism during growth and lactation. PPAR Res.

[54] Kersten S (2014). Integrated physiology and systems biology of PPARalpha. Mol Metab.

[55] Rakhshandehroo M, Sanderson LM, Matilainen M, Stienstra R, Carlberg C, de et al. Comprehensive analysis of PPARalpha-dependent regulation of hepatic lipid metabolism by expression profiling. PPAR Res. 2007;2007:26839. 10.1155/2007/26839 PMC2233741 18288265

[56] Patsouris D, Mandard S, Voshol PJ, Escher P, Tan NS, Havekes LM, et al. PPARalpha governs glycerol metabolism. J Clin Invest. 2004;114(1):94–103. 10.1172/JCI20468 PMC437964 15232616

[57] White HM, Koser SL, Donkin SS (2011). Differential regulation of bovine pyruvate carboxylase promoters by fatty acids and peroxisome proliferator-activated receptor-alpha agonist. J Dairy Sci.

[58] Way JM, Harrington WW, Brown KK, Gottschalk WK, Sundseth SS, Mansfield TA, et al. Comprehensive messenger ribonucleic acid profiling reveals that peroxisome proliferator-activated receptor gamma activation has coordinate effects on gene expression in multiple insulin-sensitive tissues. Endocrinology. 2001;142(3):1269–77. 10.1210/endo.142.3.8037 11181544

[59] Dreyer C, Krey G, Keller H, Givel F, Helftenbein G, Wahli W (1992). Control of the peroxisomal beta-oxidation pathway by a novel family of nuclear hormone receptors. Cell.

[60] Tugwood JD, Issemann I, Anderson RG, Bundell KR, McPheat WL, Green S (1992). The mouse peroxisome proliferator activated receptor recognizes a response element in the 5′ flanking sequence of the rat acyl CoA oxidase gene. EMBO J.

[61] Colca JR, McDonald WG, Waldon DJ, Leone JW, Lull JM, Bannow CA, et al. Identification of a novel mitochondrial protein (“mitoNEET”) cross-linked specifically by a thiazolidinedione photoprobe. Am J Physiol Endocrinol Metab. 2004;286(2):E252–60. 10.1152/ajpendo.00424.2003 14570702

[62] Kersten S (2005). Regulation of lipid metabolism via angiopoietin-like proteins. Biochem Soc Trans.

[63] Kharitonenkov A, Shiyanova TL, Koester A, Ford AM, Micanovic R, Galbreath EJ, et al. FGF-21 as a novel metabolic regulator. J Clin Invest. 2005;115(6):1627–35. 10.1172/JCI23606 PMC1088017 15902306

[64] Hondares E, Rosell M, Gonzalez FJ, Giralt M, Iglesias R, Villarroya F (2010). Hepatic FGF21 expression is induced at birth via PPARalpha in response to milk intake and contributes to thermogenic activation of neonatal brown fat. Cell Metab.

[65] Loor JJ, Everts RE, Bionaz M, Dann HM, Morin DE, Oliveira R, et al. Nutrition-induced ketosis alters metabolic and signaling gene networks in liver of periparturient dairy cows. Physiol Genomics. 2007;32(1):105–16. 10.1152/physiolgenomics.00188.2007 17925483

[66] Schoenberg KM, Giesy SL, Harvatine KJ, Waldron MR, Cheng C, Kharitonenkov A, et al. Plasma FGF21 is elevated by the intense lipid mobilization of lactation. Endocrinology. 2011;152(12):4652–61. 10.1210/en.2011-1425 21990311

[67] Kuo T, Chen TC, Yan S, Foo F, Ching C, McQueen A, et al. Repression of glucocorticoid-stimulated angiopoietin-like 4 gene transcription by insulin. J Lipid Res. 2014;55(5):919–28. 10.1194/jlr.M047860 PMC3995469 24565756

[68] Ryden M (2009). Fibroblast growth factor 21: an overview from a clinical perspective. Cell Mol Life Sci.

[69] Mattijssen F, Kersten S (2012). Regulation of triglyceride metabolism by Angiopoietin-like proteins. Biochim Biophys Acta.

[70] Oishi K, Tomita T (2011). Thiazolidinediones are potent inducers of fibroblast growth factor 21 expression in the liver. Biol Pharm Bull.

[71] Hegardt FG (1999). Mitochondrial 3-hydroxy-3-methylglutaryl-CoA synthase: a control enzyme in ketogenesis. Biochem J.

[72] Connor EE, Li RW, Baldwin RL, Li C (2010). Gene expression in the digestive tissues of ruminants and their relationships with feeding and digestive processes. Animal.

[73] Meertens LM, Miyata KS, Cechetto JD, Rachubinski RA (1998). Capone JPA mitochondrial ketogenic enzyme regulates its gene expression by association with the nuclear hormone receptor PPARalpha. EMBO J.

