# Supplementary material for: Level of dietary energy and 2,4-thiazolidinedione alter molecular and systemic biomarkers of inflammation and liver function in Holstein cows
Source: J Anim Sci Biotechnol. 2017 Aug 1;8:64. doi: 10.1186/s40104-017-0196-y (PMC5537929; doi:10.1186/s40104-017-0196-y)
Supplement: Additional file 1: Table S1. — GenBank accession number, sequence and amplicon size of primers used to analyze gene expression by quantitative PCR. Table S2 Gene symbol, gene name, and description of the main biological function and biological processes of the targets analyzed in subcutaneous adipose tissue and muscle. Table S3 Sequencing results obtained from PCR product. Table S4 qPCR performance among the genes measured in adipose tissue and muscle. (DOCX 197 kb) [file 40104_2017_196_MOESM1_ESM.docx]

***Management and Sampling.*** Description related to management and sampling were reported previously at Hosseini et al., ([2015](#_ENREF_6)).

***Liver Biopsy.*** Liver was sampled via puncture biopsy as described previously ([Dann et al., 2006](#_ENREF_4)) from cows under local anesthesia before the morning feeding at 14, 21, 28 and 35 d relative to diet initiation. The liver samples were snap frozen in liquid nitrogen for further analysis. After completing the biopsies, pressure was applied with sterile gauze to stop any external bleeding, the surrounding area was cleansed with sterile saline to remove blood, and the incision was closed with 3 surgical staples. Antimicrobial ointment was applied to the incision site. The wounds and the vital signs were checked on a daily base for 7 days after biopsy to avoid any infection.

# *The TZD preparation.* The 2,4-Thiazolidinedione (Cat. Number: 375004-100G, Tech., 90%; Sigma Aldrich, St. Louis, MO) was aliquot in 4.2 g in sterile, autoclaved glass bottles and sealed subsequently. Two hours before administration, 100 mL of physiological saline (0.9% Sodium Chloride Injection, USP, Rockville, MD) was injected into the bottle and placed on a Signature Multi-Tube Vortexer (Cat. Number: 58816-115, VWR, Batavia, IL) at 2000 rpm for 2 h. The 4 mg TZD/kg of BW daily was calculated based on the actual weekly BW and injected into the jugular vein using Scalp Vein Butterfly Sets starting at 2 wk after the initiation of diet for 2 additional wk. The last 2 wk of the study served as the washout period.

***RNA Extraction, Quality Assessment and cDNA synthesis.*** Adipose tissue and muscle tissues were weighed (~50 mg and ~200 mg, respectively) and immediately placed into ice-cold QIAzol Lysis Reagent (~1 and 1.2 mL, respectively; Qiagen, Valencia, CA) in a 2 ml RNase/DNase Free tubes with the O-ring, 1 bead (Qiagen: Cat. No: 69989, 5 mm) per tube was added using the Qiagen bead dispenser. The tubes were loaded into a semi-automated homogenizer and the samples were homogenized for two times 30 sec. with 1 min. incubation time on the ice. The Total RNA plus miRNA extraction was performed following the procedure recommended by Qiagen (miRNeasy Mini Kit; Cat. # 217004)*.* The advantage of the kit was to remove the genomic DNA and includ column RNA purification as well. The RNA concentration was measured with NanoDrop ND-1000 spectrophotometer (NanoDrop Technologies). The purity of RNA was assessed by ratio of optical density OD260/280, which were above 1.9 for all samples. The RNA integrity number (RIN) was assessed by electrophoretic analysis of 28S and 18S rRNA subunits using a 2100 Bioanalyzer (Agilent Technologies), and values were above 5 for all samples. A portion of the RNA was diluted to 100 ng/μL with DNase/RNase-free water for cDNA synthesis through reverse transcription PCR. cDNA was synthesized using 100 ng of RNA, 1 μL of Random Hexamer Primers (Cat. #11034731001, Roche, Pleasanton, CA), and 9 μL of DNase/RNase-free water. The mixture was incubated at 65°C for 5 min and kept on ice for 3 min. A total of 9 μL of master mix composed of 4 μL of 5X First-Strand Buffer, 1 μg of dT18 (Integrated DNA Technologies), 2 μL of 10 m*M* dNTP mix (Cat. #18427-088, Invitrogen Corp., Grand Island, NY), 0.25 μL (200 U/µL) of RevertAid Reverse Transcriptase (Cat. # EP0441, Fermentas Inc., Pittsburgh, PA), 0.125 μL (20 U/µL) of RiboLock RNase Inhibitor (Cat. #EO0381, Fermentas Inc., PA) and 1.625 μL of DNase/RNase-free water was added. The reaction was performed in an Eppendorf Mastercycler Gradient following such temperature program: 25°C for 5 min, 42°C for 60 min, and 70°C for 5 min. The cDNA was then diluted 1:4 with DNase/RNase-free water.

***Primer Design and Evaluation*.** Primers were designed and as previously described ([Bionaz and Loor, 2008](#_ENREF_2)). Briefly, primers were designed using Primer Express V3.0.2 with minimum amplicon size of 62 bp (amplicons of 100–120 bp were of superiority, if possible) and limited 3’ G + C percentage (Applied Biosystems). Primer sets were intentionally designed to fall across exon-exon junctions. Then, primers were aligned against NCBI database through BLASTN and UCSC’s COW (*Bos taurus*) Genome Browser Gateway to determine the compatibility of primers with already annotated sequence of the corresponding gene in both databases. Prior to qPCR, primers were verified through a 20-μL PCR reaction, which followed the same procedures of qPCR described below except the dissociation step. A universal reference cDNA amplified from all samples was utilized to ensure the identification of genes. Five microliters of PCR product was run in a 2% agarose gel stained with SYBER**^®^** Safe (Cat. # S33102, Life Technologies, Grand Island, NY), and the remaining 15 μL were cleaned with a QIAquick PCR Purification Kit (Cat. #28104, Qiagen) and sequenced at the Core DNA Sequencing Facility of the Roy J. Carver Biotechnology Center at the University of Illinois, Urbana (in Supplemental Table 3). The sequencing product was confirmed through BLASTN at the National Center for Biotechnology Information (NCBI) database. Only primers that presented a single band of the expected size and the right amplification product were used for qPCR. The accuracy of a pair of primers was evaluated by the presence of a unique peak during the dissociation step at the end of qPCR. The details of primer sequences and the description of genes are shown in Supplemental Tables 1 and 2.

***Quantitative PCR (qPCR)*.** qPCR was performed in a MicroAmp Optical 384-Well Reaction Plate (Cat. #4309849, Applied Biosystems, Grand Island, NY). Within each well, 4 μL of diluted cDNA combined with 6 μL of mixture composed of 5 μL 1×SYBR Green master mix (Cat. # 95073-05K, Quanta, Gaithersburg, MD), 0.4 μL each of 10 μ*M* forward and reverse primers, and 0.2 μL of DNase/RNase-free water were added. Three replicates and a 6-point standard curve plus the nontemplate control (NTC) were run for each sample to test the relative expression level. qPCR was conducted in ABI Prism 7900 HT SDS instrument (Applied Biosystems) following the conditions below: 10 min at 95°C, 40 cycles of 15 s at 95°C (denaturation), and 1 min at 60°C (annealing + extension). The presence of a single PCR product was verified by the dissociation protocol using incremental temperatures to 95°C for 15 s, then 65 °C for 15 s. The threshold cycle (Ct) data were analyzed and transformed using the standard curve with the 7900 HT Sequence Detection System Software (version 2.2.1, Applied Biosystems). Data were then normalized with the geometric mean of the three ICG as previously described by ([Vandesompele et al., 2002](#_ENREF_14)).

***Relative mRNA Abundance of Genes within Liver Tissue.*** Efficiency of qPCR amplification for each gene was calculated using the standard curve method (Efficiency = 10(–1/slope)). Relative mRNA abundance among measured genes was calculated as previously reported ([Bionaz and Loor, 2008](#_ENREF_2)), using the inverse of PCR efficiency raised to ΔCt (gene abundance = 1/EΔCt, where ΔCt = Ct of tested gene – geometric mean Ct of 3 internal control genes). Overall mRNA abundance for each gene among all samples of the same adipose tissue was calculated using the median ΔCt, and overall percentage of relative mRNA abundance was computed from the equation: 100 × mRNA abundance of each individual gene / sum of mRNA abundance of all the genes investigated (see Supplemental Table 4).

**Supplemental Table 1.** GenBank accession number, sequence and amplicon size of primers used to analyze gene expression by quantitative PCR.

| **Accession no.** | **Gene** | **Primers**^1^ | **Primers (5’-3’)** ^2^ | **(bp)** ^3^ | **Source** |
| --- | --- | --- | --- | --- | --- |
| ***Glucose hemostasis and g*luconeogenesis** | | | | | |
| XM_590552.7 | *INSR* | F.3015 | CGGAGCTCAGAGATCACGACTAT | 106 | ([Naeem et al., 2012](#_ENREF_12)) |
|  |  | R.3120 | AGGTTCACAGTTAAGTGCTCAGATGA |  |  |
| XM_003581871.1 | *IRS1* | F.4268 | TGTTGACTGAACTGCACGTTCT | 112 | ([Ji et al., 2012](#_ENREF_7)) |
|  |  | R.4379 | CATGTGGCCAGCTAAGTCCTT |  |  |
| NM_174737.2 | *PCK1* | F.601 | AAGATTGGCATCGAGCTGACA | 120 | ([Chen, 2013](#_ENREF_3)) |
|  |  | R.720 | GTGGAGGCACTTGACGAACTC |  |  |
| XM_005226995.1 | *PC* | F.3497 | GCAAGGTCCACGTGACTAAGG | 124 | ([Chen, 2013](#_ENREF_3)) |
|  |  | R.3620 | GGCAGCACAGTGTCCTGAAG |  |  |
| ***Ketogenesis*** | | | | | |
| NM_001045883.1 | *HMGCS2* | F.1229 | TCTGGCCCATCACTCTGCC | 126 | ([Kaufmann et al., 2012](#_ENREF_8)) |
|  |  | R.1354 | AGTGGGGAGCCTGGAGAAGC |  |  |
| ***Lipid metabolism nuclear receptors*** | | | | | |
| NM_001034036.1 | *PPARA* | F.729 | CATAACGCGATTCGTTTTGGA | 102 | ([Graugnard, 2011](#_ENREF_5)) |
|  |  | R.830 | CGCGGTTTCGGAATCTTCT |  |  |
| NM_001083636.1 | *PPARD* | F.1295 | TGTGGCAGCCTCAATATGGA | 100 | ([Loor et al., 2007](#_ENREF_10)) |
|  |  | R.1376 | GACGGAAGAAGCCCTTGCA |  |  |
| ***Lipid metabolism*** | | | | | |
| NM_174693.2 | *DGAT1* | F.190 | CCACTGGGACCTGAGGTGTC | 101 | ([Bionaz and Loor, 2008](#_ENREF_2)) |
|  |  | R.290 | GCATCACCACACACCAATTCA |  |  |
| NM_001035289.3 | *ACOX1* | F.944 | CCATTGCCGTCCGATACAGT | 99 | ([Loor et al., 2007](#_ENREF_10)) |
|  |  | R.1042 | GTTTATATTGCTGGGTTTGATAATCCA |  |  |
| NM_174494.2 | *ACADVL* | F.707 | CCAGCCCCTGTGGAAAATACTA | 62 | ([Thering et al., 2009](#_ENREF_13)) |
|  |  | R.768 | GCCCCCGTTACTGATCCAA |  |  |
| ***Hepatokines*** | | | | | |
| NM_001046043.2 | *ANGPTL4* | F.697 | AGGAAGAGGCTGCCCAAGAT | 109 | ([Loor et al., 2007](#_ENREF_10)) |
|  |  | R.805 | CCCTCTCTCCCTCTTCAAACAG |  |  |
| XM_002695200.1 | *FGF21* | F.514 | CAGAGCCCCGAAAGTCTCTTG | 126 | ([Khan et al., 2014](#_ENREF_9)) |
|  |  | R.639 | AAAGTGCAGCGATCCGTACAG |  |  |
| ***Cytokines and inflammatory mediator*** | | | | | |
| NM_173966.3 | *TNF* | F.367 | CCAGAGGGAAGAGCAGTCCC | 114 | ([Mukesh et al., 2010](#_ENREF_11)) |
|  |  | R.480 | TCGGCTACAACGTGGGCTAC |  |  |
| ***Internal control genes*** | | | | | |
| NM_001034034.2 | *GAPDH* | F.275 | TGGAAAGGCCATCACCATCT | 53 | ([Bionaz and Loor, 2007](#_ENREF_1)) |
|  |  | R.327 | CCCACTTGATGTTGGCAG |  |  |
| NM_001101152.2 | *RPS9* | F.128 | CCTCGACCAAGAGCTGAAG | 64 | ([Bionaz and Loor, 2007](#_ENREF_1)) |
|  |  | R.191 | CCTCCAGACCTCACGTTTGTTC |  |  |
| NM_001037471.2 | *UXT* | F.300 | TGTGGCCCTTGGATATGGTT | 101 | ([Bionaz and Loor, 2007](#_ENREF_1)) |
|  |  | R.400 | GGTTGTCGCTGAGCTCTGTG |  |  |

^1^Primer direction (F – forward; R – reverse) and hybridization position on the sequence.

^2^ Primer sequence

^3^ Amplicon size in base pair (bp)

**Supplemental Table 2.** Gene symbol, gene name, and description of the main biological function and biological processes of the targets analyzed in subcutaneous adipose tissue and muscle.

| Symbol | Name | Cellular Localization | Summary description from NCBI |
| --- | --- | --- | --- |
| *ACADVL* | Acyl-CoA dehydrogenase, very long chain | Mitochondrial | Active toward esters of long-chain and very long chain fatty acids such as palmitoyl-CoA, mysritoyl-CoA and stearoyl-CoA. |
| *ACOX1* | Acyl-CoA oxidase 1, palmitoyl | Peroxisomal | Catalyzes the desaturation of very long chain acyl-CoAs to 2-trans-enoyl-CoAs. |
| *ANGPTL4* | Angiopoietin-like 4 | Extracellular space | May act as a regulator of angiogenesis and modulate tumorgenesis. Inhibits proliferation, migration, and tubule formation of endothelial cells and reduces vascular leakage. |
| *DGAT1* | Diacylglycerol O-acyltransferase homolog 1 | ER membrane | Catalyzes the terminal and only committed step in triacylglycerol synthesis by using diacylglycerol and fatty acyl CoA as substrates. In contrast to DGAT2 it is not essential for survival. May be involved in VLDL (very low density lipoprotein) assembly. |
| *FGF21* | Fibroblast growth factor 21 | Extracellular Space | Stimulates glucose uptake in differentiated adipocytes via the induction of glucose transporter SLC2A1/GLUT1 expression. |
| *HMGCS2* | 3-hydroxy-3-methylglutaryl-CoA synthase 2 (mitochondrial) | Mitochondrial | It catalyzes the first reaction of ketogenesis, a metabolic pathway that provides lipid-derived energy for various organs during times of carbohydrate deprivation, such as fasting. |
| *INSR* | Insulin receptor | Plasma membrane | Binding of insulin to the insulin receptor stimulates glucose uptake. |
| *IRS1* | Insulin receptor substrate 1 | Cytosol | This gene encodes a protein which is phosphorylated by insulin receptor tyrosine kinase. |
| *PC* | Pyruvate carboxylase | Mitochondrial | This gene encodes pyruvate carboxylase, which requires biotin and ATP to catalyse the carboxylation of pyruvate to oxaloacetate. The active enzyme is a homotetramer arranged in a tetrahedron which is located exclusively in the mitochondrial matrix. Pyruvate carboxylase is involved in gluconeogenesis, lipogenesis, insulin secretion and synthesis of the neurotransmitter glutamate. |
| *PCK1* | Phosphoenolpyruvate carboxykinase 1 | Cytosol and Mitochondrial | This gene is a main control point for the regulation of gluconeogenesis. The cytosolic enzyme encoded by this gene, along with GTP, catalyzes the formation of phosphoenolpyruvate from oxaloacetate, with the release of carbon dioxide and GDP. The expression of this gene can be regulated by insulin, glucocorticoids, glucagon, cAMP, and diet. |
| *PPARA* | Peroxisome proliferator-activated receptor alpha | Nucleus | PPARalpha is a member of the nuclear receptor family of ligand-activated transcription factors that heterodimerize with the retinoic X receptor (RXR) to regulate gene expression |
| *PPARD* | Peroxisome proliferator-activated receptor delta | Nucleus | Receptor that binds peroxisome proliferators such as hypolipidemic drugs and fatty acids. Regulates the peroxisomal beta-oxidation pathway of fatty acids. Functions as transcription activator for the acyl-CoA oxidase gene |
| *TNF* | Tumor necrosis factor alpha | Nucleus | It is mainly secreted by macrophages and can induce cell death of certain tumor cell lines. It is potent pyrogen causing fever by direct action or by stimulation of interleukin-1 secretion and is implicated in the induction of cachexia, Under certain conditions it can stimulate cell proliferation and induce cell differentiation |

**Supplemental Table 3.** Sequencing results obtained from PCR product.

| **Gene** | **Sequence** |
| --- | --- |
| ***ACADVL*** | GCAAGATTTGGATCAGTAACGGGGG |
| ***ACOX1*** | GGTNTTCNGTAACNACCCGGGGGAACCAGAACCACAGATTTTGGATTATCAAACCCAGCAATAGTAAACAAG |
| ***ANGPTL4*** | CTGCACAGACTGCCCAGGGACTGCCAGGAGCTGCTTTGAAGAGGGAGCTGAATACGGATCGCTGCACATT |
| ***DGAT1*** | CGCAGCGATCCCTGTTCAGTTCTGACAGTGGCTTCAGCAACTACCGTGGCATCCTGAATTGGTGTGTGGTGATGCACA |
| ***FGF21*** | ATTCAGATCTTGGGAGTTAAAACATACCAGGGTTTCTCTGCCAGGGGCCAGATGGGACAG |
| ***GAPDH*** | ATCTTCCAGGAGCGAGATCCTGCCAACATCAAGTGGG |
| ***HMGCS2*** | TCTCTTAGTGCATCGTGGTTTGGCAGCAGTTTCTTTTCTTTCCGGGTGTCCCAGGATGCTTCTCCAGGCTCCCCAC |
| ***INSR*** | CATGACTTCCTTCGTGACTTCAACGCAGGTGTTCCATTGGCCTATTTGACTTGTCATCTGAGCACTTAACTGTGAACCTTAAA |
| ***IRS1*** | ATCAGGCAGAAAAGCACTGTGACACCAGAACAATGAGTCTGCATAAACTTCATCTTCAACCTTAAGGACTTAGCTGGCCAACATGGAA |
| ***PC*** | GATCATAGGAGTACAGAACTCATCTGGAAGAATCGAGTGACCACGCTGAGACTGGCAGCCTGACCATCCCCGACCCCTGCCTTCAGGACACTGTGCTGCCAGA |
| ***PCK1*** | GCCATGTGTACAGCAGTCGCATCATGACGAGGATGGGCACCAGCGTCCTGGAAGCGCTGGGGGACGGCGAGTTCGTCAAGTGCCTCCACAAA |
| ***PPARA*** | AATTGAAGGCAGAAATCCTTACGTGTGAGCATGACCTAGAAGATTCCGAAACCGCGGA |
| ***PPARD*** | TTCTGTGCGGAGACCGGCCAGGCCTCAAGTATCGGTTCTCAGGTGGAGGCAACG |
| ***RSP9*** | GCCAGTATGGGCTCCGGAACAAACGTGAGGTCTGGAGG |
| ***TNF*** | TCACTCTCCGGGGCAGCTCCGGTGGTGGGACTCGTATGCCAATGCCC  TCATGGAA |
| ***UXT*** | CTGGTTATCGTGGAGCTCTCAGTTCATTGATCGTAAGAGCAGTCTCCTCACAGAGCTCAGCGACAATCTCAT |

**Supplemental Table 4.** qPCR performance among the genes measured in adipose tissue and muscle.

| **Gene** | **Median Ct^1^** | **Median ∆Ct^2^** | **Slope^3^** | **(R^2^)^4^** | **Efficiency^5^** |
| --- | --- | --- | --- | --- | --- |
| ***ACADVL*** | 22.43 | -0.52 | -4.21 | 0.99 | 1.73 |
| ***ACOX1*** | 21.26 | -1.64 | -3.56 | 0.98 | 1.91 |
| ***ANGPTL4*** | 27.15 | 4.15 | -2.97 | 0.99 | 2.17 |
| ***DGAT1*** | 25.59 | 2.70 | -3.16 | 0.98 | 2.07 |
| ***FGF21*** | 28.94 | 4.58 | -3.40 | 0.99 | 1.97 |
| ***HMGCS2*** | 20.14 | -2.67 | -3.12 | 1.00 | 2.09 |
| ***INSR*** | 25.37 | 2.63 | -2.95 | 0.98 | 2.18 |
| ***IRS1*** | 24.51 | 1.72 | -3.21 | 0.98 | 2.05 |
| ***PC*** | 24.44 | 1.62 | -3.53 | 0.98 | 1.92 |
| ***PCK1*** | 21.22 | -1.61 | -3.45 | 0.99 | 1.95 |
| ***PPARA*** | 23.66 | 0.77 | -3.08 | 1.00 | 2.11 |
| ***PPARD*** | 28.67 | 5.68 | -3.07 | 0.98 | 2.12 |
| ***TNF*** | 29.04 | 6.01 | -3.03 | 1.00 | 2.14 |

| ^1^ The median is calculated considering all time points and all cows. |
| --- |
| ^2^ The median of ∆Ct is calculated as [Ct gene – geometrical mean of Ct internal controls] for each time point and each steer. |
| ^3^ Slope of the standard curve. |
| ^4^ R^2^ stands for the coefficient of determination of the standard curve. |
| ^5^ Efficiency is calculated as [10^(-1 / Slope)^] |

**References**

Bionaz, M. and J. J. Loor. 2007. Identification of reference genes for quantitative real-time PCR in the bovine mammary gland during the lactation cycle. Physiological genomics 29(3):312-319.

Bionaz, M. and J. J. Loor. 2008. Gene networks driving bovine milk fat synthesis during the lactation cycle. BMC genomics 9:366.

Chen, S. 2013. Effects of close-up dietary energy strategy and prepartal dietary monensin on hepatic mrna expression of enzymes involved in glucose and lipid metabolism in transition cows. Page 67. Vol. Master. University of Illinois.

Dann, H. M., N. B. Litherland, J. P. Underwood, M. Bionaz, A. D'Angelo, J. W. McFadden, and J. K. Drackley. 2006. Diets during far-off and close-up dry periods affect periparturient metabolism and lactation in multiparous cows. J Dairy Sci 89(9):3563-3577.

Graugnard, D. E. 2011. Immune function, gene expression, blood indices and performance in transition dairy cows affected by diet and inflammation. Page 198 in Animal Science. Vol. PhD. University of Illinois.

Hosseini, A., M. R. Tariq, F. Trindade da Rosa, J. Kesser, Z. Iqbal, O. Mora, H. Sauerwein, J. K. Drackley, E. Trevisi, and J. J. Loor. 2015. Insulin Sensitivity in Adipose and Skeletal Muscle Tissue of Dairy Cows in Response to Dietary Energy Level and 2,4-Thiazolidinedione (TZD). PloS one 10(11):e0142633.

Ji, P., J. S. Osorio, J. K. Drackley, and J. J. Loor. 2012. Overfeeding a moderate energy diet prepartum does not impair bovine subcutaneous adipose tissue insulin signal transduction and induces marked changes in peripartal gene network expression. J Dairy Sci 95(8):4333-4351.

Kaufmann, L. D., F. Dohme-Meier, A. Munger, R. M. Bruckmaier, and H. A. van Dorland. 2012. Metabolism of grazed vs. zero-grazed dairy cows throughout the vegetation period: hepatic and blood plasma parameters. Journal of animal physiology and animal nutrition 96(2):228-236.

Khan, M. J., C. B. Jacometo, D. E. Graugnard, M. N. Correa, E. Schmitt, F. Cardoso, and J. J. Loor. 2014. Overfeeding Dairy Cattle During Late-Pregnancy Alters Hepatic PPARalpha-Regulated Pathways Including Hepatokines: Impact on Metabolism and Peripheral Insulin Sensitivity. Gene regulation and systems biology 8:97-111.

Loor, J. J., R. E. Everts, M. Bionaz, H. M. Dann, D. E. Morin, R. Oliveira, S. L. Rodriguez-Zas, J. K. Drackley, and H. A. Lewin. 2007. Nutrition-induced ketosis alters metabolic and signaling gene networks in liver of periparturient dairy cows. Physiological genomics 32(1):105-116.

Mukesh, M., M. Bionaz, D. E. Graugnard, J. K. Drackley, and J. J. Loor. 2010. Adipose tissue depots of Holstein cows are immune responsive: inflammatory gene expression in vitro. Domestic animal endocrinology 38(3):168-178.

Naeem, A., J. K. Drackley, J. Stamey, and J. J. Loor. 2012. Role of metabolic and cellular proliferation genes in ruminal development in response to enhanced plane of nutrition in neonatal Holstein calves. J Dairy Sci 95(4):1807-1820.

Thering, B. J., M. Bionaz, and J. J. Loor. 2009. Long-chain fatty acid effects on peroxisome proliferator-activated receptor-alpha-regulated genes in Madin-Darby bovine kidney cells: optimization of culture conditions using palmitate. J Dairy Sci 92(5):2027-2037.

Vandesompele, J., K. De Preter, F. Pattyn, B. Poppe, N. Van Roy, A. De Paepe, and F. Speleman. 2002. Accurate normalization of real-time quantitative RT-PCR data by geometric averaging of multiple internal control genes. *Genome Biology* 3(7):research0034-research0034.0011
